# Supplementary material for: Enhanced Cataluminescence Sensor Based on SiO2/MIL-53(Al) for Detecting Isobutylaldehyde
Source: Molecules. 2024 Jul 11;29(14):3287. doi: 10.3390/molecules29143287 (PMC11279756; doi:10.3390/molecules29143287)
Supplement: Supplementary file 1 [file molecules-29-03287-s001.zip › molecules-3053337-supplementary.pdf]

Molecules

Electronic Supporting Material

Enhanced Cataluminescence Sensor Based on SiO_2/MIL-53(Al) for Detecting Isobutylaldehyde

Qianchun Zhang [1,*], Xixi Long [1], Shan Tang [1], Li Jiang [1], Zhaoru Ban [1], Yanju Chen [1] and Runkun Zhang [2,*]

[1] Key Laboratory for Analytical Science of Food and Environment Pollution of Qian Xi Nan, School of Biology and Chemistry, Xingyi Normal University for Nationalities, Xingyi 562400, China; longxixi@xynun.edu.cn (X.L.); tangshan@xynun.edu.cn (S.T.); jiangli@xynun.edu.cn (L.J.); banzhaoru@xynun.edu.cn (Z.B.); chenyanju@xynun.edu.cn (Y.C.)

[2] Guangdong Provincial Engineering Research Center of Public Health Detection and Assessment, School of Public Health, Guangdong Pharmaceutical University, Guangzhou 510310, China

* Correspondence: zhangqianchun@xynun.edu.cn (Q.Z.); zhangrk@gdpu.edu.cn (R.Z.); Tel.: +86-589-3296359 (Q.Z.)

Table S1. Textural properties of SiO_2, MIL-53(Al), and SiO_2/MIL-53(Al).

Materials	Surface area (m^2/g)	Average pore size (nm)	Pore volume (cm^3/g)
SiO_2	3.81	9.2035	0.008775
MIL-53(Al)	304.03	15.9499	1.604335
SiO_2/MIL-53(Al)	138.92	8.8267	0.306555

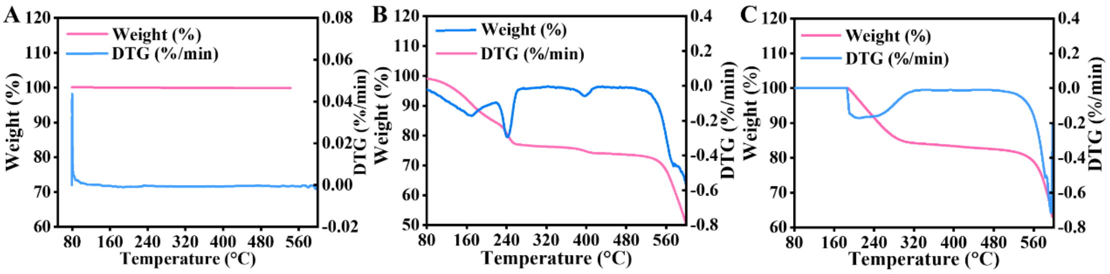

Figure S1. Thermogravimetric (TG) analysis for (A) SiO_2, (B) MIL-53(Al), and (C) SiO_2/MIL-53(Al) samples. In the air atmosphere, from 80 °C to 600 °C, the heating rate is 10 °C/min.

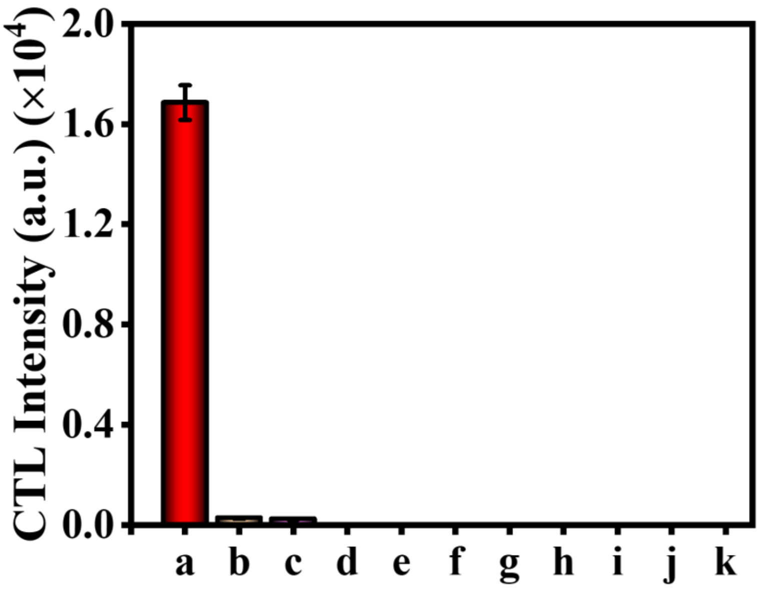

Figure S2. CTL response of diverse gases on SiO_2/MIL-53(Al): a, IBL; b, 2-butoxy ethanol; c, styrene; d, methyl isobutyl ketone; e, 3-methyl-2-butene-1-ol; f, cycloheptanone; g, formamide; h, chloropropene; i, tetrachloroethylene; j, cholamine; k, sec-butyl alcohol.

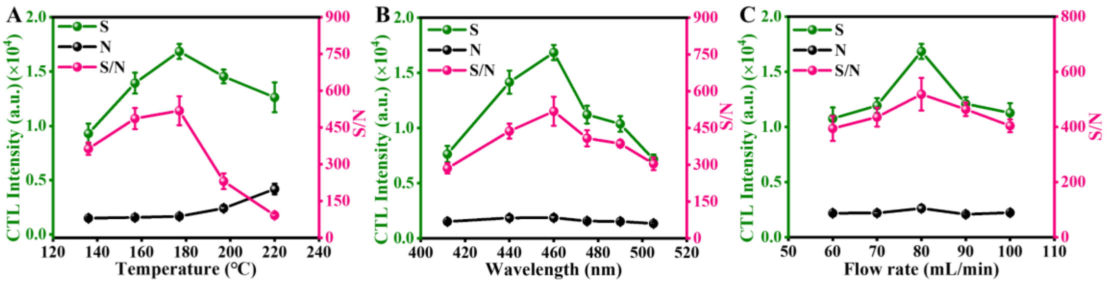

Figure S3. (A) CTL intensity, S/N, and background noise (N) are influenced by the operating temperature (wavelength, 460 nm; flow rate, 80 mL min^{-1}). (B) wavelength dependence of the CTL intensity, S/N, and background noise(N) (flow rate, 80 mL min^{-1}; temperature, 177 °C) (C) flow rate dependence of the CTL intensity, S/N, and background noise(N) (wavelength, 460 nm; temperature, 177 °C). The injection volume was1 mL at a concentration of 155 ppm, *n* = 5. Error bars represent ±SD (standard deviation).

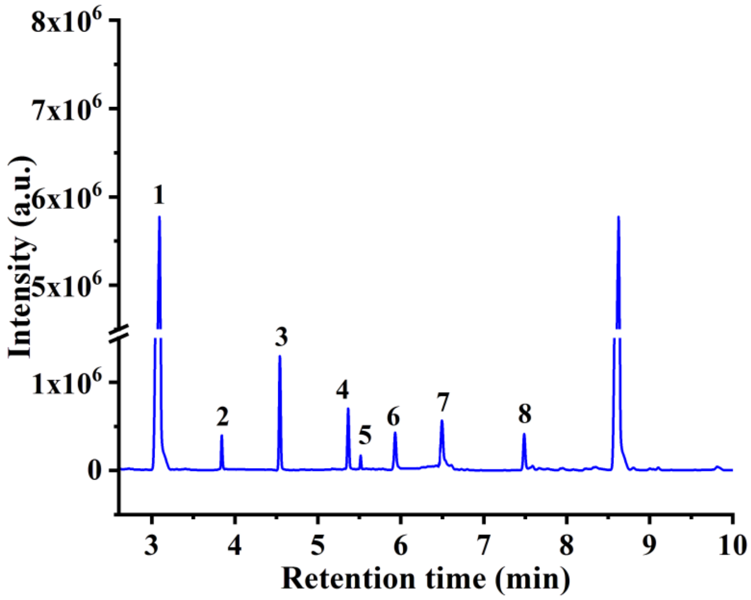

Figure S4. GC-MS chromatograms of tail gas samples. The peaks are: 1. Carbon dioxide, 2. HCHO, 3. CH_3CHO, 4. CH_3CH_2CHO, 5. $CH_2=C(CH_3)CHO$, 6. CH_3COCH_3,7. $H_3CCH(CH_3)CHO$, and 8. $(CH_3)_2CHCOOH$.

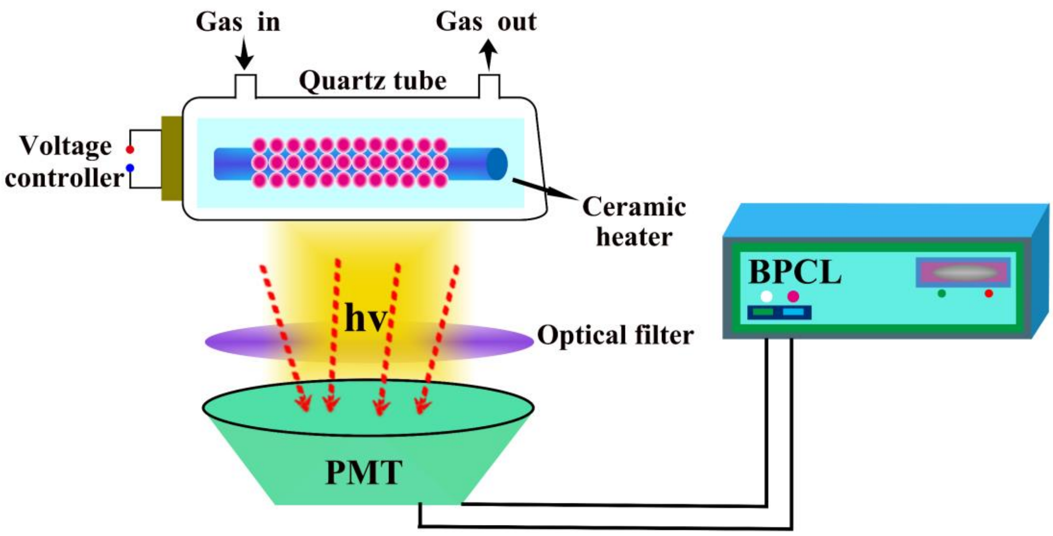

Figure S5. SiO_2/MIL-53(Al) CTL sensing system device.
